# Supplementary material for: Incidence of hemoparasitic infections in cattle from central and northern Thailand
Source: PeerJ. 2022 Aug 10;10:e13835. doi: 10.7717/peerj.13835 (PMC9375545; doi:10.7717/peerj.13835)
Supplement: Supplemental Information 1 — - [file peerj-10-13835-s001.docx]

**Supplementary data**

**Table S1** Multiple infections in cattle from central and northern Thailand

| **Hemoparasites infection** | **Frequency** | **%** |
| --- | --- | --- |
| **Two pathogen infection** | | |
| *B. bovis* and *T. orientalis* | 7 | 0.66 |
| *B. bovis* and *A. marginale* | 1 | 0.09 |
| *T. orientalis* and *A. marginale* | 278 | 26.08 |
| **Three pathogen infection** | | |
| *B. bovis, T. orientalis,* and *A. marginale* | 5 | 0.47 |
| **Total** | 291 | 27.30 |
