# Supplementary figures and images for: Incidence of hemoparasitic infections in cattle from central and northern Thailand
Source: PeerJ. 2022 Aug 10;10:e13835. doi: 10.7717/peerj.13835 (PMC9375545; doi:10.7717/peerj.13835)

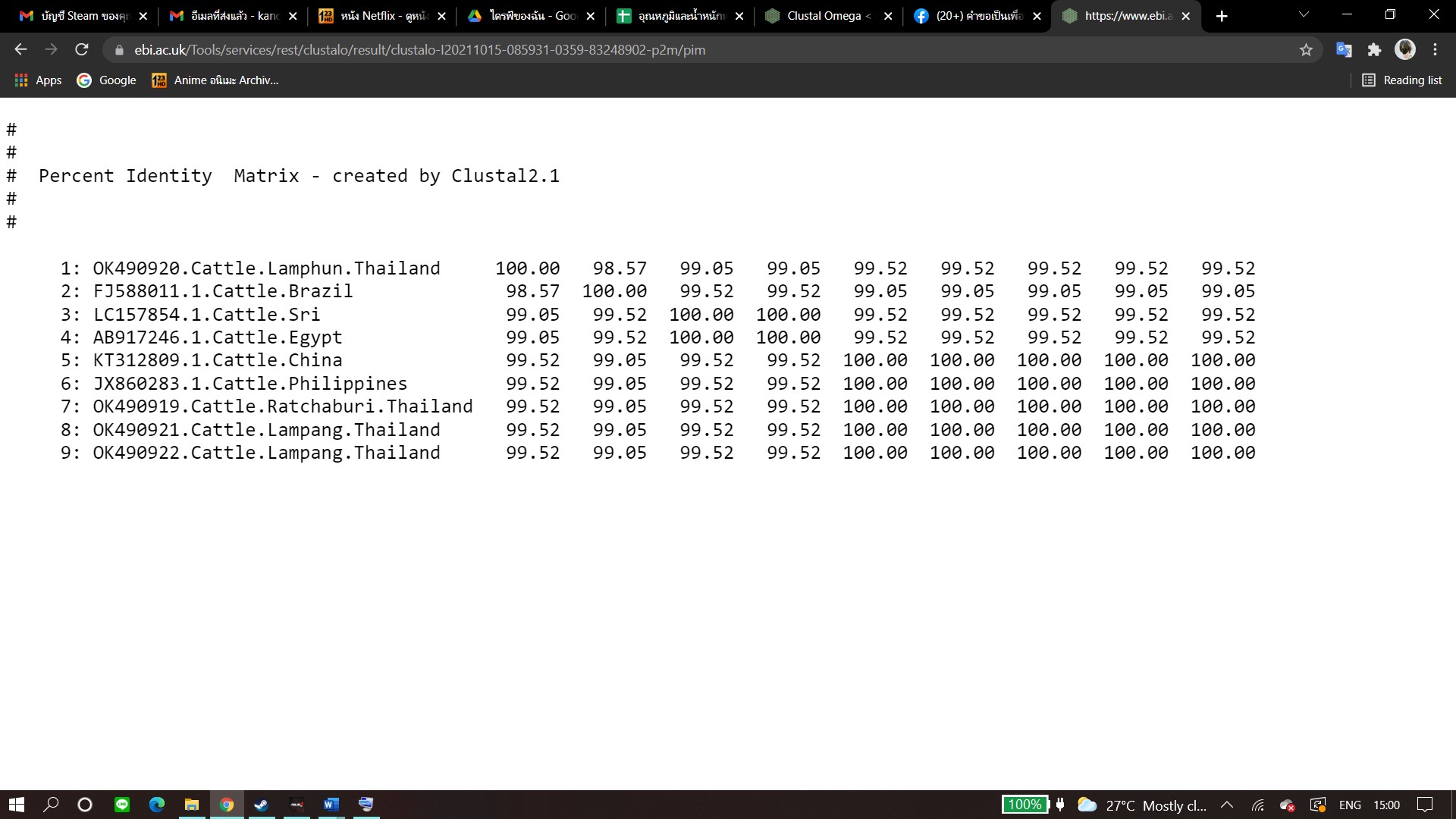

Supplement: Supplemental Information 2 — - [file peerj-10-13835-s002.jpg]

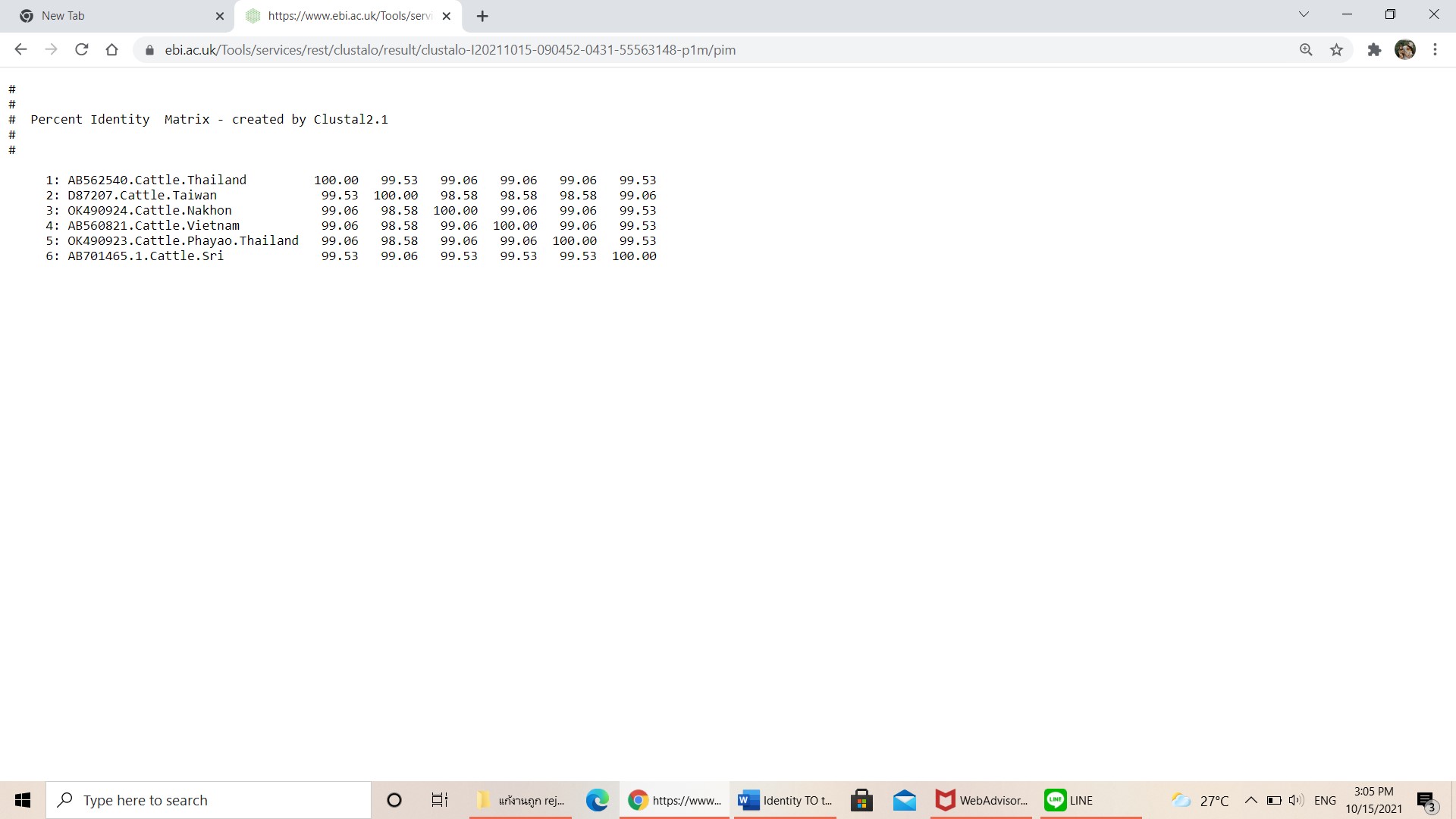

Supplement: Supplemental Information 3 — - [file peerj-10-13835-s003.jpg]

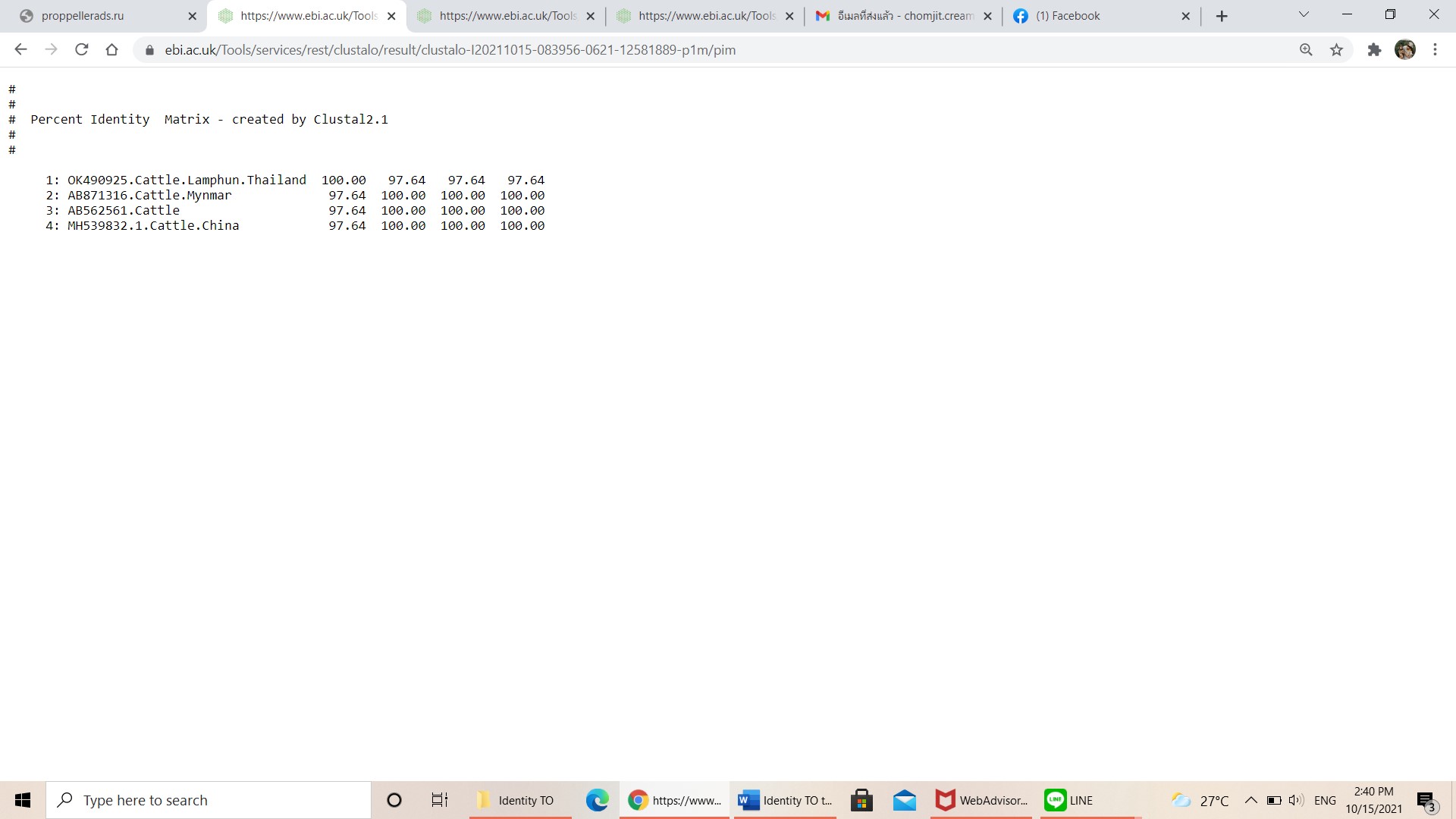

Supplement: Supplemental Information 4 — - [file peerj-10-13835-s004.jpg]

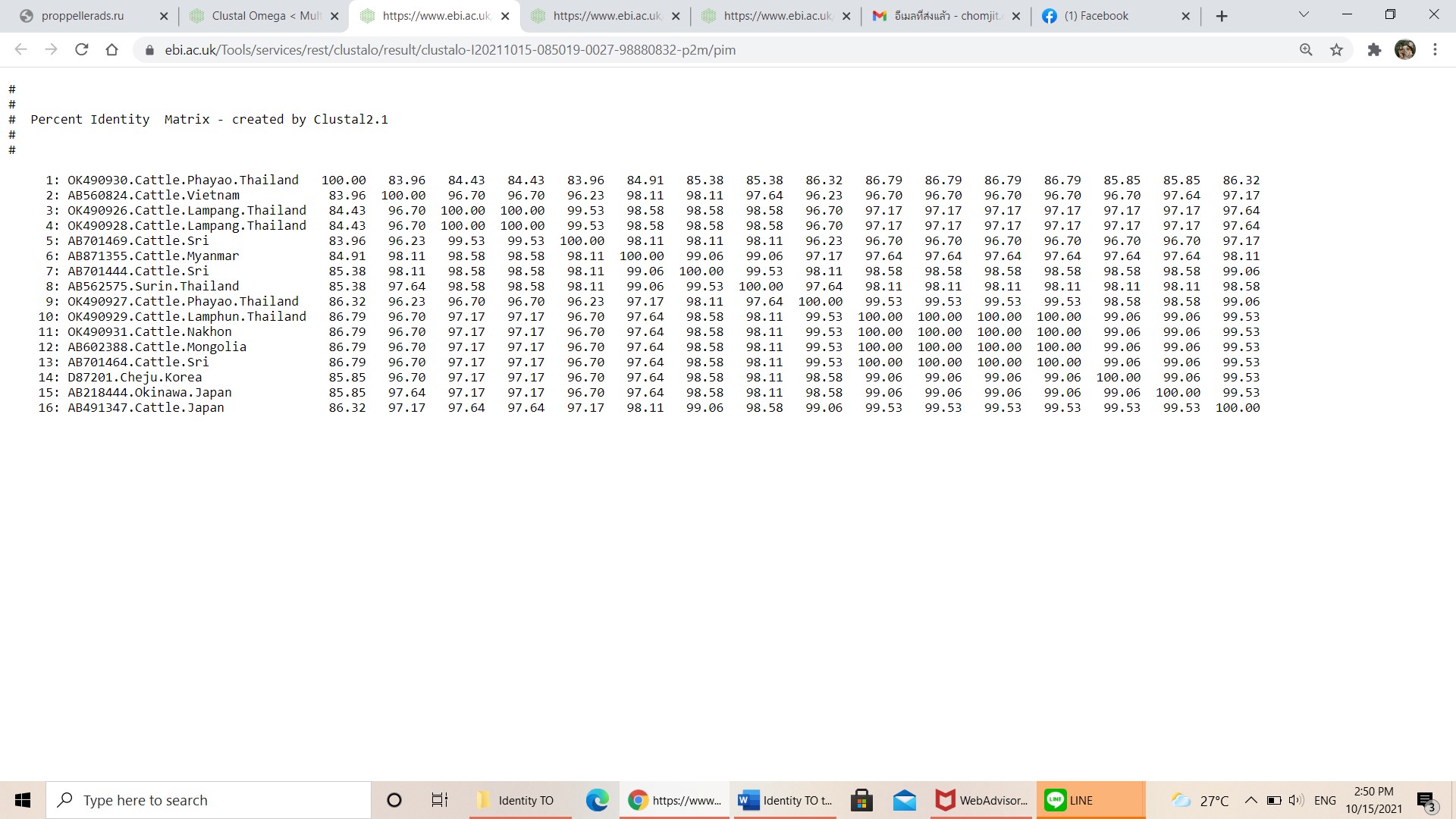

Supplement: Supplemental Information 5 — - [file peerj-10-13835-s005.jpg]

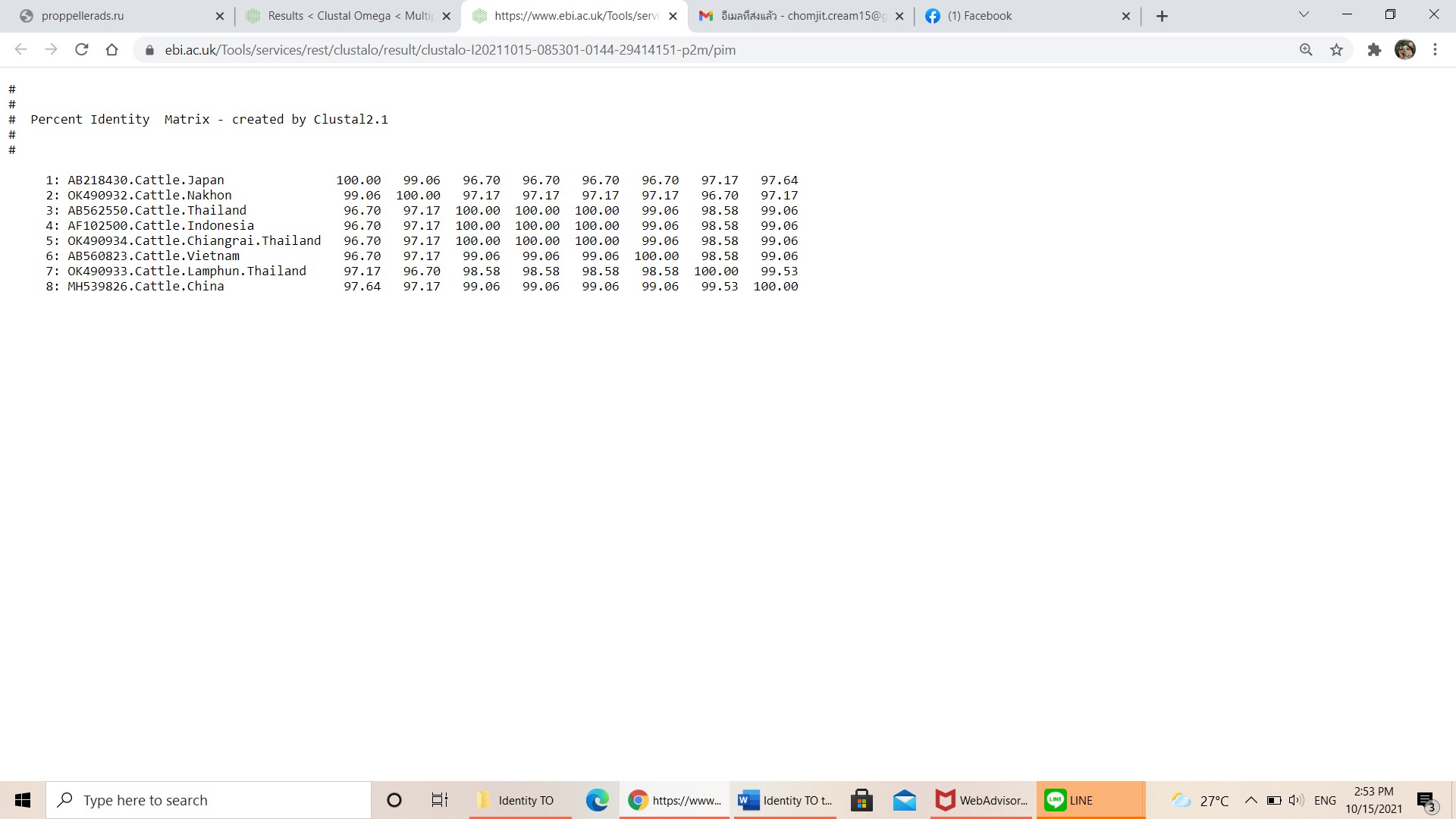

Supplement: Supplemental Information 6 — - [file peerj-10-13835-s006.jpg]

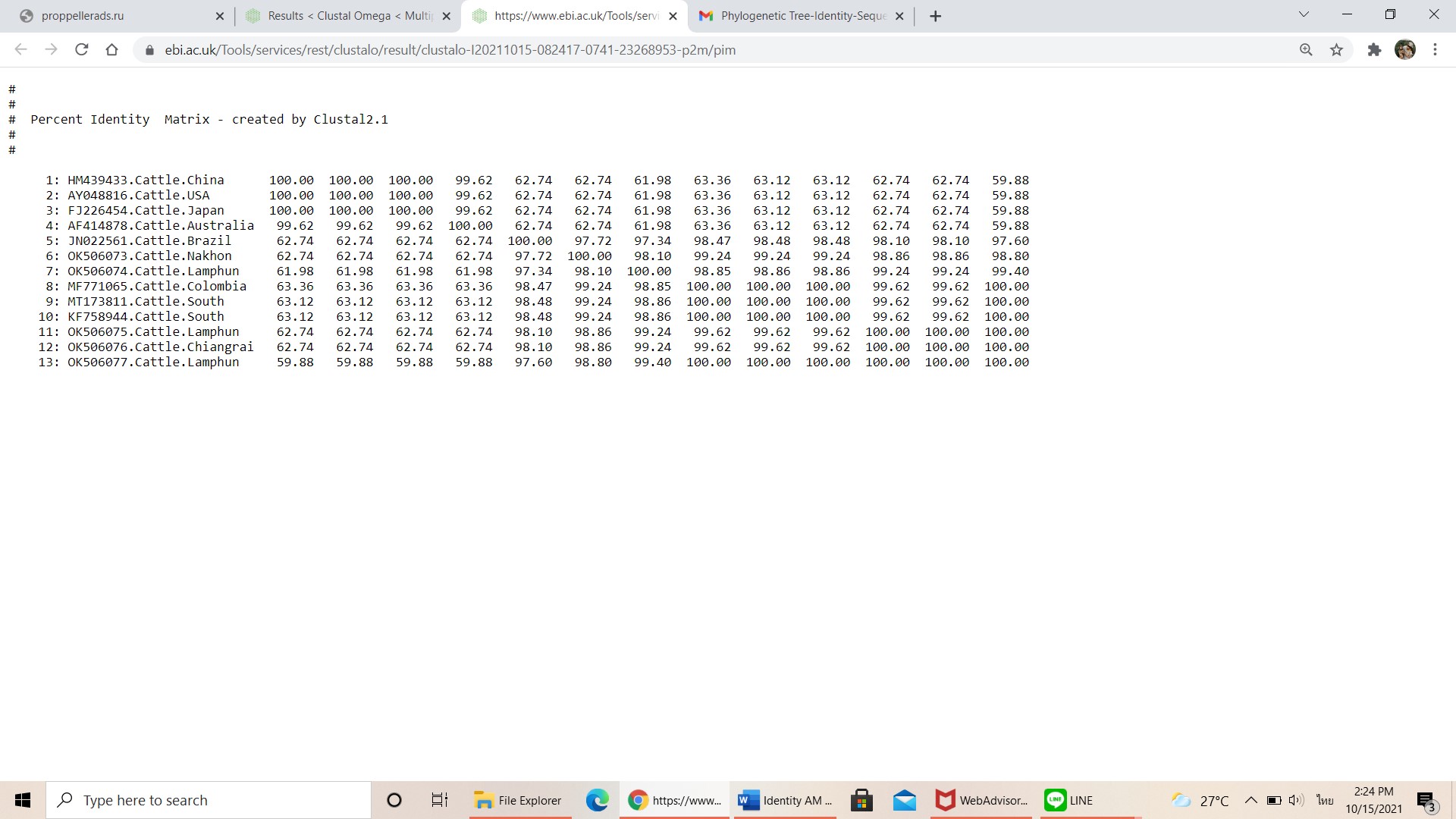

Supplement: Supplemental Information 7 — - [file peerj-10-13835-s007.jpg]
