# Supplementary material for: Incidence of hemoparasitic infections in cattle from central and northern Thailand
Source: PeerJ. 2022 Aug 10;10:e13835. doi: 10.7717/peerj.13835 (PMC9375545; doi:10.7717/peerj.13835)
Supplement: Supplemental Information 8 [file peerj-10-13835-s008.docx]

>OK490919.Cattle.Nakhon Pathom.Thailand

ACAGATTCAGCTTCACTACAAAGATATTCAGCCGTCGTATTAGGCAAACATTGAGTGATATCATCAGGTGGAATGTTCCTGAAGATTTTGAAGAAAGGAGCATCGAACGTATCACTCAACTTACTAGCAGCTACGAAGATTACATGTTGACCCAGATTCCAACTCTTTCCAAGTTTGCACGTCGTTATGCTGACATGGTGAAGAAGGTTC

>OK490920.Cattle.Lamphun.Thailand

ACAGATTCAGCTTCACTACAAAGATATTCAGCCGTCGTATTAGGCAAACATTGAGTGATATCATCAGGTGGAATGTTCCTGAAGATTTTGAAGAAAGGAGCATCGAACGTATCACTCAACTTACTAGCGGCTACGAAGATTACATGTTGACCCAGATTCCAACTCTTTCCAAGTTTGCACGTCGTTATGCTGACATGGTGAAGAAGGTTC

>OK490921.Cattle.Lampang.Thailand

ACAGATTCAGCTTCACTACAAAGATATTCAGCCGTCGTATTAGGCAAACATTGAGTGATATCATCAGGTGGAATGTTCCTGAAGATTTTGAAGAAAGGAGCATCGAACGTATCACTCAACTTACTAGCAGCTACGAAGATTACATGTTGACCCAGATTCCAACTCTTTCCAAGTTTGCACGTCGTTATGCTGACATGGTGAAGAAGGTTC

>OK490922.Cattle.Lampang.Thailand

ACAGATTCAGCTTCACTACAAAGATATTCAGCCGTCGTATTAGGCAAACATTGAGTGATATCATCAGGTGGAATGTTCCTGAAGATTTTGAAGAAAGGAGCATCGAACGTATCACTCAACTTACTAGCAGCTACGAAGATTACATGTTGACCCAGATTCCAACTCTTTCCAAGTTTGCACGTCGTTATGCTGACATGGTGAAGAAGGTTC

>OK490923.Cattle.Phayao.Thailand

TGCCAACGACGTCGTTTTTTCTGCCTCGGAGGGATATCGTTTCAAGACTCTTAAGGTTGGAGATAAAACTTTGTATACCGTTGATACATCCAAATTCACTCCAACCGTTGCCCACAGAATTAAGCATGGTGATGCCTTGTTCTTCAAGCTTGACCTTTCCCATGCCAAGCCACTCTTGTTCAAGAAGAAGACTGACAAGGATTGGGTTCAGT

>OK490924.Cattle.Nakhon Pathom.Thailand

CGGCCCCCCCCGGTGAAAATTTTACAGTCAATGCAACCAATGCCAACGACGTCGTTTTTACTGCCTCGGAGGGATATCGTTTCAAGACTCTTAAGGTTGGAGATAAAACTTTGTATACCGTTGATTCATCCAAATTCACTCCAACCGTTGCCCACAGAATTAAGCATGGTGATGCCTTGTTCTTCAAGCTTGACCTTTCCCATGCCAAGCCACTCTTGTTCAAGAAGAAGACTGACAAGGATTGGGTTCAGTTTAACTTTGGCCAGTACCTTGACGAATTTGTATGGAAGGAAAAGAAGGAACTCAAGGATCTAGATGCATCCAAGTTTGCAGAGGCAGGTCTTTTTGCAGCTGATACATTCGGTACTGGTAAGGTTTATGACTTTGTCGGACCCTTCAAGGTTAAGAGTGTTAAGTTTGAGAATAACGTGGTTGGTGACCCTAAAAAGGCCAAATACACTGCAGTCAAAGTATACGTAGGTACCGATGATAAGAAAGTAGTAAGACTTGACTACTTCTACACTGCTGATGAGAGATTCAAGGAGGTTTACTTCAAATTGGTAGATGGAAAATGGAAAAAGCTTGAGCAGAGCGAGGCAAACAAGGATTTGCACGCTATGAACAATGCTTGGCCTTTGGACTACAAGCCTCTTGTCGACAAGTTCTCCAC

>OK490925.Cattle.Lamphun.Thailand

TCCCAACGACGTCGTTTTTACTGCCAATGATGGATTCCGCTTCAAGACACTTAAGGTTGGAGATAAAACATTGTATACCGTTGATACATCCAAATTCACTCCAACAGTCGCTCACAGACTTAAGCATGGTGAAAGCTTGTTCTTCAAGCTCGATCTTTCCCATGCCAAACCACTCTTGTTCAAGAAGAAGACTGACAAGGATTGGGTTCAGT

>OK490926.Cattle.Lampang.Thailand

ACCCGATGACGTCGTTTTTACTGCTAATGATGGATATCGCTTCAAGACTCTCAAAGTTGGAGATAAAACATTGTATACTGTTGATACAACCAAATTCACTCCAACAGTCGCCCACAGACTTAAGCATGGTGATGCATTGTTCTTCAAGCTTGAACTTTCTCCTGCCAAGCCACTTTTGTTCAAAATGAAGTCGGACAAGGAATGGGTTCAGT

>OK490927.Cattle.Phayao.Thailand

ACCCGATGACGTCGTTTTTACTGCTAATGATGGATATCGTTTCAAGACTCTCAAAGTTGGAGATAAAACATTGTATACCGTTGATACATCCAAATTCACTCCAACAGTTGCCCACAGACTTAAGCATGGTGATGCATTGTTCTTCAAGCTTGACCTTTCTCATGCCAAGCCACTCTTGTTCAAAATGAAGTCGGACAAGGAATGGGTTCAGT

>OK490928.Cattle.Lampang.Thailand

ACCCGATGACGTCGTTTTTACTGCTAATGATGGATATCGCTTCAAGACTCTCAAAGTTGGAGATAAAACATTGTATACTGTTGATACAACCAAATTCACTCCAACAGTCGCCCACAGACTTAAGCATGGTGATGCATTGTTCTTCAAGCTTGAACTTTCTCCTGCCAAGCCACTTTTGTTCAAAATGAAGTCGGACAAGGAATGGGTTCAGT

>OK490929.Cattle.Lamphun.Thailand

ACCCGATGACGTCGTTTTTACTGCTAATGATGGATATCGTTTCAAGACTCTCAAAGTTGGAGATAAAACATTGTATACCGTTGATACATCCAAATTCACTCCAACAGTCGCCCACAGACTTAAGCATGGTGATGCATTGTTCTTCAAGCTTGACCTTTCTCATGCCAAGCCACTCTTGTTCAAAATGAAGTCGGACAAGGAATGGGTTCAGT

>OK490930.Cattle.Phayao.Thailand

ACCAAATGACTTCTTTTTTCCTGCTAATGATGGTTCCGCTTTCAAGACTCTCAGGTTGGGAGATAAACCATTGTATACCGTTGATACTCCCAATTTCACTCCACCAGTCCCCCACAAACTTAACCATGGTGATGCCTTGTTCTTCAAGCTTGACCTTTCTCATGCCAAGCCCTTCTTGTTCAAAATGAAATCGGACAAGGAAGGGGTTCATT

>OK490931.Cattle.Nakhon Pathom.Thailand

ACCCGATGACGTCGTTTTTACTGCTAATGATGGATATCGTTTCAAGACTCTCAAAGTTGGAGATAAAACATTGTATACCGTTGATACATCCAAATTCACTCCAACAGTCGCCCACAGACTTAAGCATGGTGATGCATTGTTCTTCAAGCTTGACCTTTCTCATGCCAAGCCACTCTTGTTCAAAATGAAGTCGGACAAGGAATGGGTTCAGT

>OK490932.Cattle.Nakhon Pathom.Thailand

CGCCAACGACGTCGTTTTTACTGTTGAGGACGGATACCGCTTCAAGACTCTCAAGGTTGGAGATAAGACCCTGTATACCGTAGATACATCCAAATTCACTCCAACTGTCGCCCACAGACTGAAGCATGATGAAGACCTGTTCTTCAAGCTCAACCTGTCCCACGCCAAGCCCCTTCTGTTCAAGAAGAAGAGCGACAAGGATTGGGTACAGT

>OK490933.Cattle.Lamphun.Thailand

CGCCAACGACGTCGTTTTTACTGCCGAGGAGGGATACCGCTTCAAGACTCTCAAGGTTGGAGATAAGACCCTGTATACCGTAGATACATCCAAATTCACTCCAACCGTCGCCCACAGAATGAAGCATGCTGAAGACCTGTTCTTCAAGCTCAACCTGTCCCACGCCAAGCCCCTTCTGTTCAAGAAGAAGACCGACAAGGATTGGGTACAGT

>OK490934.Cattle.Chiangrai.Thailand

CGCCAACGACGTCGTTTTTACTGCTGAGGAGGGATACCGCTTCAAGACTCTCAAGGTTGGAGATAAGACCCTGTATACTGTAGATACATCCAAATTCACTCCAACCGTCGCCCACAGAATGAAGCATGCTGAAGACCTGTTCTTCAAGCTCAACCTGTCCCACGCCAAGCCCCTTCTGTTCAAGAAGAAGAGCGACAAGGATTGGGTACAGT

>OK506073.Cattle.Nakhon Pathom. Thailand

GACATATCTTTCGACTTACTGAGACCAATTACTTCGTCTTCAAAATTGATGAAATCAAAACACCTCAGTCATGTTAAATGGCTGCTATGACGTGCTGCACACAGATTTGCCTGTGTCCCCGTATGTATGTGCCGGGATAGGCGCAAGCTTTGTTGACATCTCTAAGCAAGTAACCACAAAGCTGGCCTACAGGGGCAAGGTTGGGATTCCAGTTTACTCCGGAAATATCCTTGGGGCAGGTGGGTTCTACCACGGGCTATTTGATGAGTCT

> OK506074.Cattle.Lamphun. Thailand

GCTATTACCCGCGACTTACTGAGACCAATTACTTCGTAGTCAAAATTGATGAAATCAAAACACCTCAGTCATGTTAAATGGCTGCTATGACGTGCTGCACACACATTTGCCTGTGTCCCCGTATGTATGTGCCGGGATAGGCGCAAGCTTTGTTGACATCTCTAAGCAAGTAACCACAAAGCTGGCCTACAGGGGCAAGGTTGGGATTCCAGTTTACTCCGGAAATATCCTTGGGGCAGGTGGGTTCTACCACGGGCTATTTGATGACTCG

> OK506075.Cattle.Lamphun. Thailand

GCTATTACCCGCGACTTACTGAGACCAATTACTTCGTAGTCAAAATTGATGAAATCAAAACACCTCAGTCATGTTAAATGGCTGCTATGACGTGCTGCACACAGATTTGCCTGTGTCCCCGTATGTATGTGCCGGGATAGGCGCAAGCTTTGTTGACATCTCTAAGCAAGTAACCACAAAGCTGGCCTACAGGGGCAAGGTTGGGATTCCAGTTTACTCCGGAAATATCCTTGGGGCAGGTGGGTTCTACCACGGGCTATTTGATGACTCT

> OK506076.Cattle.Chiangrai. Thailand

GCTATTACCCGCGACTTACTGAGACCAATTACTTCGTAGTCAAAATTGATGAAATCAAAACACCTCAGTCATGTTAAATGGCTGCTATGACGTGCTGCACACAGATTTGCCTGTGTCCCCGTATGTATGTGCCGGGATAGGCGCAAGCTTTGTTGACATCTCTAAGCAAGTAACCACAAAGCTGGCCTACAGGGGCAAGGTTGGGATTCCAGTTTACTCCGGAAATATCCTTGGGGCAGGTGGGTTCTACCACGGGCTATTTGATGACTCT

> OK506077.Cattle.Lamphun. Thailand

GCTATTACCCGCGACTTACTGAGACCAATTACTTCGTAGTCAAAATTGATGAAATCAAAACACCTCAGTCATGTTAAATGGCTGCTATGACGTGCTGCACACAGATTTGCCTGTGTCCCCGTATGTATGTGCCGGGATAGGCGCAAGCTTTGTTGACATCTCTAAGCAAGTAACCACAAAGCTGGCCTACAGGGGCAAGGTTGGGATTCCAGTTTACTCCGGAAATATCCTTGGGGCAGGTGGGTTCTACCACGGGCTATTTGATGAGTCT
